# Feeding Induced by Cannabinoids Is Mediated Independently of the Melanocortin System

**DOI:** 10.1371/journal.pone.0002202

**Published:** 2008-05-21

**Authors:** Puspha Sinnayah, Erin E. Jobst, Joseph A. Rathner, Angela D. Caldera-Siu, Luciana Tonelli-Lemos, Aaron J. Eusterbrock, Pablo J. Enriori, Emmanuel N. Pothos, Kevin L. Grove, Michael A. Cowley

**Affiliations:** 1 Division of Neuroscience, Oregon National Primate Research Center, Oregon Health & Science University, Portland, Oregon, United States of America; 2 School of Physical Therapy, Pacific University, Hillsboro, Oregon, United States of America; 3 Neurological Science Institute, Beaverton, Oregon, United States of America; 4 Department of Pharmacology and Experimental Therapeutics and Program in Neuroscience, Tufts University School of Medicine, Boston, Massachusetts, United States of America; 5 Department of Physiology, Faculty of Medicine, Monash University, Clayton, Victoria, Australia; University of Granada, Spain

## Abstract

**Background:**

Cannabinoids, the active components of marijuana, stimulate appetite, and cannabinoid receptor-1 (CB1-R) antagonists suppress appetite and promote weight loss. Little is known about how CB1-R antagonists affect the central neurocircuitry, specifically the melanocortin system that regulates energy balance.

**Methodology/Principal Findings:**

Here, we show that peripherally administered CB1-R antagonist (AM251) or agonist equally suppressed or stimulated feeding respectively in A^y^ , which lack a functional melanocortin system, and wildtype mice, demonstrating that cannabinoid effects on feeding do not require melanocortin circuitry. CB1-R antagonist or agonist administered into the ventral tegmental area (VTA) equally suppressed or stimulated feeding respectively, in both genotypes. In addition, peripheral and central cannabinoid administration similarly induced c-Fos activation in brain sites suggesting mediation via motivational dopaminergic circuitry. Amperometry-detected increases in evoked dopamine (DA) release by the CB1-R antagonist in nucleus accumbens slices indicates that AM251 modulates DA release from VTA terminals.

**Conclusions/Significance:**

Our results demonstrate that the effects of cannabinoids on energy balance are independent of hypothalamic melanocortin circuitry and is primarily driven by the reward system.

## Introduction

Obesity is a major health epidemic in developed nations. Obesity has been implicated in the etiologies of both type 2 diabetes and cardiovascular disease [1]. Recently, a number of drugs have entered clinical trials as therapies for obesity. A novel class of drug undergoing clinical trials are the cannabinoid receptor-1 (CB1-R) antagonists, such as Rimonabant (Accomplia, sanofi-aventis, SR141716). Cannabinoids, the active components of marijuana, stimulate appetite and feeding and CB1-R antagonists reduce feeding in animals and humans [2], [3]. Although Rimonabant is in late-stage clinical trials as a therapy for obesity and metabolic syndrome, little is known about how CB1-R antagonists affect the central neurocircuitry that regulates energy balance.

Central melanocortin pathways play a pivotal role in regulating appetite and energy balance. Proopiomelanocortin (POMC) neurons in the arcuate nucleus of the hypothalamus (ARH) produce the peptide α-MSH which binds to and activates melanocortin-4 receptors (MC4-R) [4], causing reduced food intake and increased energy expenditure. MC4-R strongly regulate body weight and appetite in humans and other species [4]. Central antagonism of MC4-R increases feeding and obesity [5], [6], whereas central administration of MTII, a potent non-selective melanocortin agonist, suppresses appetite [7].

Recent work has demonstrated that the CB1-R antagonist AM251-an analogue of Rimonabant-increases GABAergic inhibition onto POMC neurons *in vitro* [8]. This effect would be expected to increase appetite by decreasing the likelihood of release of the anorectic peptide α-MSH. However, this is contrary to the known anorectic action of cannabinoid antagonists. This paradox suggests that the appetite-suppressing effects of the CB1-R antagonist are mediated outside of the ARH. Thus, an alternate possibility is that CB-1 R antagonists suppress appetite via effects on central reward pathways.

Cannabinoids clearly exert effects on reward and motivation. The nucleus accumbens (NAc), a region involved in reward, is an important mediator of appetite [9]. The ventral tegmental area (VTA) of the midbrain/pons is a major source of dopamine onto neurons in the NAc, and palatable food consumption has been linked to dopamine release within the NAc [10]. Systemic cannabinoid administration increases the firing rate of dopaminergic neurons in the VTA, an effect blocked by CB1-R antagonists [11], [12]. Central administration of the CB1-R agonist WIN 55,212-2 decreases GABA-mediated inhibitory postsynaptic currents onto dopaminergic VTA neurons [13]. Thus, it appears that cannabinoids increase dopaminergic neuronal activity within the VTA by decreasing inhibitory input onto these cells. Consistent with the hypothesis that dopaminergic activity in the VTA regulates feeding, it has recently been reported that ghrelin increases the firing rate of dopaminergic VTA neurons and that ghrelin injected into the VTA increases feeding in rats [14].

To investigate whether the melanocortin system is involved in central cannabinoid effects on appetite, we studied the effects of a CB1-R antagonist AM251 and a non-selective agonist WIN 55,212-2 on feeding behavior in agouti yellow (A^y^) mice. A^y^ mice over-express Agouti protein, an endogenous inverse agonist of MC3 and MC4 receptors, offering a convenient model of melanocortin antagonism. This study reports that feeding behavior mediated by the cannabinoid system is independent of the melanocortin system and that dopaminergic reward pathways likely play a fundamental role in cannabinoid-induced feeding.

## Methods

### Animal care and housing

All animal procedures were approved by the Oregon National Primate Research Center Institutional Animal Care and Use Committee. Adult male C57BL/6J mice (WT) and Agouti B6.Cg-A^y^/J (A^y^; Jackson Labs, Bar Harbor, ME) were individually housed under a 12 h light/dark cycle and constant temperature (23°C). Standard chow and water were available *ad libitum* except where noted.

### Drugs

All drugs were freshly prepared on day of use. AM251 (Tocris) and WIN 55,212-2 (Tocris) were dissolved in 10% dimethyl sulfoxide (DMSO) and sterile nonpyrogenic 0.9% NaCl. Drugs were administered intraperitoneally (IP) in a volume of 0.1±0.02 mL (according to body weight) for feeding tests. Control mice received vehicle in a corresponding volume containing 10% DMSO and sterile saline.

### Electrophysiological recording from POMC neurons

Coronal hypothalamic slices containing the arcuate nucleus (ARH) were prepared from 8 week-old male POMC-EGFP mice as described previously [15]. This procedure was in accordance with the National Institutes of Health and the American Association for Accreditation of Laboratory Animal Care guidelines and was approved by Oregon Health & Science University. Briefly, mice were anesthetized with isoflurane and killed by decapitation. The brain was quickly removed and cooled in ice-cold artificial cerebrospinal fluid (ACSF) solution of the following composition (in mM): 126 NaCl, 2.5 KCl, 1.2 MgCl_2_/6H_2_O, 2.4 CaCl_2_/2H_2_O, 1.2 NaH_2_PO_4_/H_2_O, 21.4 NaHCO_3_ and 11.1 glucose (saturated with 95% O_2_/5% CO_2_). A block of hypothalamic tissue containing the ARH was dissected and coronal slices (185 µm) were cut with a vibrating slicer (Leica VT1000S). Slices were stored for at least 1 h in a holding chamber with ACSF at room temperature and continuously bubbled with 95% O_2_/5% CO_2_. Individual slices were submerged in a recording chamber and superfused continuously with carbogenated ACSF at 35°C (3–5 mL/min). To record IPSCs, excitatory currents were blocked with TTX (1 µM; Sigma), D-AP5 (25 µM;Tocris) and CNQX (10 µM; Tocris). To record EPSCs, inhibitory currents were blocked with TTX (1 µM; Sigma) and picrotoxin (10 µM; Tocris).

Recordings were made from arcuate POMC neurons, identified by bright green fluorescence [15]. Electrode resistances were 2–4 MΩ when filled with an intracellular solution of the following composition, in mM: IPSCs: 70 K- gluconate, 5 HEPES, 0.1 EGTA, 57 KCl, 1.5 MgCl_2_, 2 (Mg)ATP, 0.5 (Na)GTP; EPSCs: 132 K-gluconate, 4 NaCl, 0.5 CaCl_2,_. 10 HEPES, 5 EGTA-free, 4 (Mg)ATP, 0.5 (Na)GTP. Whole cell voltage clamp configuration (Axopatch 200B, Axon Instruments, CA) was used. Data were filtered at 2 kHz and then sampled at 50–100 kHz by pClamp 8.2 software (Axon Instruments). Data were analyzed using Minianalysis (Synaptosoft, Inc., GA).

### Hypothalamic peptide secretion

#### Static incubation of hypothalamic explants

Twenty four C57BL/6J ∼6 wk-old mice were used. α-MSH release assays was performed as previously described [16]. Briefly, 2-mm thick hypothalamic slices taken from the base of the brain (to include the PVH and ARH) were incubated in artificial cerebrospinal fluid (aCSF) for 45 min, followed by a 45 min incubation in aCSF (n =  8 hypothalami), or 3 uM CB1 agonist-WIN55212-2 (n = 8 hypothalami) or 3 uM CB1 antagonist AM251(n = 8 hypothalami). Tissue viability was verified by a further incubation in aCSF containing 56 mM KCl.

#### Radioimmunoassay

α-MSH radioimmunoreactivity (RIA) was measured as previously described [16]. The lowest detectable level that could be distinguished from the zero standard was 0.31 fmol/tube. The intra assay variation (CV%) was determined by replicate analysis (n  = 8) of two samples at α-MSH concentrations of 2 and 10 fmol/tube, and the results were 9.4% and 8.2% respectively. The inter assay variation (CV%) was 10.3% and 11.2% for the range of value measured.

### Physiological studies

Individually housed mice were habituated to the behavioral testing procedure by daily IP injections of 0.1 mL sterile saline and overnight fasting every third day for at least 2 weeks prior to treatment. Young male (6–8 weeks) A^y^ (n = 14) and WT (n = 14) C57BL/6J mice were age- and weight-matched and randomly assigned to treatment groups. To study the effects of WIN 55,212-2, mice were not fasted prior to IP injection in the morning. To determine the effects of AM251, mice were fasted overnight prior to treatment and given *ad libitum* access to a predetermined amount of food immediately after IP injection with AM251 (5 mg/kg) or saline vehicle. Food intake was recorded at 1, 2, 4, 8, and 24 hour intervals and normalized to wild-type saline control.

### VTA cannulation-surgical preparation

Adult (6–8 weeks) WT (n = 8) and A^y^ (n = 8) mice were anesthetized with pentobarbital (50 mg/kg, IP). The head of the mouse was shaved and aligned in a stereotaxic apparatus (Kopf, CA) such that lambda-bregma plane was horizontal. The head was cleaned with betadine and an incision made with a sterile scalpel. A hole was made according to the following co-ordinates for VTA: 3.5 mm caudal, 0.4 mm lateral and 3.0 mm ventral [17]. These co-ordinates placed the guide cannula (30gauge, 3 mm; Plastics Inc.) just dorsal to the VTA, allowing injections with minimal damage to the VTA. The cannula was fixed in place with acrylic dental cement and two anchoring skull screws. To maintain patency, a stylet protruding 0.5 mm past the tip of the guide cannula was inserted. After surgery, mice were allowed to recover for one week, when food intake and body weight returned to pre-surgery levels. Mice were handled daily and habituated to the injection procedure for 2 weeks prior to experimental procedures. Mice were fasted overnight prior to the morning injection of AM251 (1 µg). Mice were held gently, while the stylet was removed and an injector was inserted. The injector was 1 mm longer than the guide cannula. Preliminary experiments in which dye was injected into the VTA identified an ideal spread of injection to be 1 µl. Injections (1 µl) were administered slowly over one minute via a Hamilton syringe and mice were placed in home cages. The injector was left in the brain for an additional minute before removal. The stylet was replaced and food intake was recorded at 1, 2, 4, 8, and 24 hour intervals and normalized to wild-type saline control. For intra-VTA WIN 55,212-2 (1 µg) administration, mice were not fasted overnight prior to day of injection and the above mentioned procedure was carried out. For intra-VTA saline control administration, mice were injected with a corresponding volume containing 10% DMSO and sterile saline and is specified in text as saline for simplicity. To determine the effects of intra-VTA injections of AM251 on IP cannabinoid agonist-induced food intake, non-fasted WT mice received intra-VTA injections of saline (Sal) or AM251 (1 µg) followed by IP administration of saline or WIN 55,212-2 (1 mg/kg) 15 minutes later. Conversely, to determine the effects of intra-VTA injections of WIN 55,212-2 on IP cannabinoid antagonist-induced attenuation of food intake, fasted WT mice received intra-VTA injections of saline (Sal) or WIN 55,212-2 (1 µg) followed by IP administration of saline or AM251 (5 mg/kg) 15 minutes later. Food intake was recorded at 1, 2, 4, 8, and 24 hour intervals and normalized to saline/saline control. Animals were perfused with formaldehyde and cannulae placement were histologically verified at end of experiments. A small number of animals (n = 3) for each genotype were found to have injection sites outside of the VTA and these were classified as VTA-missed injections. We achieved site-specific cannulations 72% of the time for the VTA.

### c-Fos immunoreactivity

Male A^y^ and WT mice were perfused with 4% paraformaldehyde 90 min after IP injection with saline-DMSO (n = 3), WIN 55,212-2 (1 mg/kg; n = 3) or AM251 (5 mg/kg; n = 4). In this study, food was not returned to fasted animals after WIN 55,212-2 treatment. Another group of male A^y^ and WT mice was perfused 90 min after intra-VTA injection with saline-DMSO (n = 3), WIN 55,212-2 (1 µg; n = 3) or AM251 (1 µg; n = 4). Brains were removed and 25 µm coronal sections were cut in sets of 4 adjacent series. Free floating sections were rinsed in 0.05 M potassium PBS (KPBS), preincubated in blocking buffer, consisting of KPBS containing 0.4% Triton-X100 (KPBSX) and 2% donkey serum, for 1 hour. One complete set of sections was incubated in c-Fos primary rabbit antibody (Santa Cruz Biotechnology, CA; 1∶20,000) in blocking buffer for 48 h. Sections were then incubated with biotinylated donkey anti-rabbit antibody (Jackson Immunoresearch, West Grove, PA; 1∶600) followed by avidin-biotin application (Vectastain, Vector Laboratories, Burlingame, CA). c-Fos IR was visualized using a chromagen label enhanced nickel chloride. Tissue sections were mounted on gelatin-coated glass slides, counterstained with cresyl violet and coverslipped.

For immunohistochemistry of c-Fos and EGFP, detection of c-Fos was carried out as described above in POMC-EGFP mice. Following washes after chromagen detection, sections were incubated in rabbit anti-GFP antibody (1∶10,000) overnight at 4°C. Sections were then incubated with fluorescein-conjugated donkey anti-rabbit secondary antibody (Jackson Immuno Research, 1∶200) in 1 M KPBS for 2 hours. Sections were then washed, mounted and coverslipped using Fluoromount-G (Southern Biotech).

For double-labeling of c-Fos and orexin or MCH, two complete sets of sections were used. One set was incubated with c-Fos antibody and a goat polyclonal antibody (Santa Cruz Biotechnology, sc-8070; 1∶5000) directed against the C-terminus of the human orexin-A peptide. The other set was incubated with c-Fos antibody and a chicken polyclonal antibody (Bachem, T-1506; 1∶3000) directed against the full length 18-residue human MCH peptide for 1 hour at room temperature followed by 48 hr at 4°C. Detection of c-Fos was carried out as described above. Following washes after chromagen detection, sections were incubated with fluorescein-conjugated donkey anti-goat or rhodamine-conjugated donkey anti-chicken fluorescent secondary antibodies (Jackson Immuno Research, 1∶200) in 1 M KPBS for 2 hours. Sections were then washed, mounted and coverslipped using Fluoromount-G (Southern Biotech).

#### c-Fos quantification

Depending on treatment, 9–11 brain regions and 4–6 sections for each region were examined using light microscopy (Nikon Eclipse E800 series microscope. Images were captured using a CoolSnapHQ camera system and Metamorph software (using 10Xobjective). Captured images were subsequently analyzed using Adobe Photoshop software, whereby a grid was placed over regions of interest and the number of labeled cells within a standardized area was counted manually by an investigator blind to the treatment group. A neuron was classified as c-Fos-positive when the nucleus was intensely stained black in colour and appeared either round or oval. Values were expressed as the mean number of c-Fos positive cells per section for each region. Regions analyzed were anatomically matched across all animals.

The number of c-Fos, EGFP-positive cells or double-labeled (c-Fos and EGFP) cells were counted from 5 sections spanning the ARH, using both light microscopy for c-Fos and fluorescence for EGFP. Values are shown as the percentage of EGFP positive cells expressing c-Fos.

The number of orexin or MCH neurons or double-labeled (orexin/c-Fos or MCH/c-Fos) cells were counted from 4–6 sections spanning the LH, using both light microscopy for c-Fos and fluorescence for orexin or MCH. Values are shown as the percentage of orexin neurons expressing c-Fos.

### Slice Recordings: Carbon fiber amperometry

Sprague Dawley male rats (3–5 weeks) were anesthetized with ketamine (200 mg/kg IP)/xylazine (20 mg/kg IP). Upon removal, the brain was immediately placed into ice-cold carbogenated (95% O_2_, 5% CO_2_) dissection solution containing (in mM): 210 sucrose, 3.5 KCl, 1 CaCl_2_ dihydrate, 4 MgCl_2_ hexahydrate, 1.25 NaH_2_PO_4_ hydrate, 10 glucose, and 26 NaHCO_3_. Coronal slices (300 µm) containing the nucleus accumbens, were cut using a vibratome. Following 1 hr recovery in carbogenated ACSF (in mM, 124 NaCl, 2.0 KCl, 1.25 KH_2_PO_4_, 2.0 MgSO_4_, 25 NaHCO_3_, 1.0 CaCl_2_, 11 glucose [pH 7.3]), each slice was transferred into a recording chamber maintained at 37°C. Slices were continually perfused with ACSF (1 ml/min). Carbon fiber amperometry was used to record the release of neurotransmitter. A disk carbon fiber electrode, 5 µm in diameter, was placed in the posterior nucleus accumbens shell at a depth of ∼50 µm. The reference electrode (Ag/AgCl wire) was inserted into the ACSF bath. Voltage was set to +700 mV (Axopatch 200B.). The bipolar stimulating electrode (diameter 0.005 inch—MS 303/3, Plastics One, Inc.) was placed within a distance of 200 µm from the carbon fiber electrode. A constant monophasic current stimulus (delivered by Isoflex stimulus isolator, AMPI Inc., controlled by Grass Instruments S88 Stimulator) was applied through the bipolar stimulating electrode with the following stimulation parameters: single rectangular pulse for 2 msec, +500 µA amplitude. For each slice, five single pulses were given in the nucleus accumbens shell at five-minute intervals while in the ACSF bath, then the slice was washed in 3 µM AM251 (in ACSF) for 30 min. After the wash, five more single pulses in five-minute intervals were given at the same location. Amperometric electrode recordings were monitored and quantified by a locally written routine on the Superscope II platform (GW Instruments). Data was acquired at 50 kHz and digitally postfiltered at 1 kHz. Background-subtracted cyclic voltammograms calibrate the electrodes and identify the released substance as DA. Mean of signal amplitudes, widths, and number of molecules in ACSF were compared to those following the 3 µM AM251 wash by 1-way ANOVA.

### Statistical Analysis

For electrophysiological data, paired Student's t test was used for comparison of two groups: 10 min of stable baseline with 10 min after treatment. The F-test was used to compare differences in the slopes derived for interevent interval and amplitude analysis. For α-MSH IR, data were analyzed by 1-way ANOVA, followed by Bonferroni's Multiple Comparison Test. For physiological analysis, data are presented as fraction of control and analysed by ANOVA. For c-Fos immunohistochemistry, two-way ANOVA was used to examine genotype versus treatment effects. For double-labeling study, counted cells were expressed as percentage of orexin positive c-Fos cells and significance between treatment groups was calculated via 2-way ANOVA. Data were expressed as mean±SEM, p<0.05 was considered significant.

## Results

### AM251 increases the frequency of inhibitory postsynaptic currents onto POMC neurons

**Figure 1:**
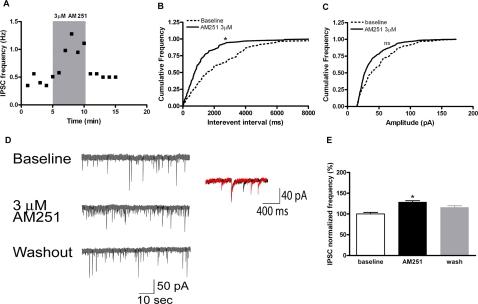
Effect of AM251 on POMC neurons. *A*, AM251 increased the IPSC frequency onto POMC neurons: time-effect plot of IPSC frequency from a representative cell in presence of AM251 (3 µM; shaded region); *B*, Cumulative plot of interevent interval or *C*, IPSC amplitude from the same cell as *A*. *D*, Examples of raw data showing IPSCs before, during and after bath application of AM251 (3 µM; left) with representative kinetics of IPSC (right inset) and conductances before (black) and during peak antagonist effect (red). *E*, Summary of effect of AM251 on normalized IPSC frequency. (* p <0.05, when compared with baseline; ns,not significant).

To directly examine the role of cannabinoid agents on arcuate melanocortin circuitry, we examined the effect of AM251 (3 µM) and WIN 55,212-2 (3 µM) on POMC neurons using POMC-EGFP mice [15]. In patch-clamp recordings from visually identified arcuate POMC neurons, the CB1-R antagonist AM251 increased the frequency of GABAergic inhibitory postsynaptic currents (IPSCs) onto POMC neurons (41%, 1.7±1.1 and 2.4±1.3 Hz, baseline and AM251, respectively; n = 7, p<0.05, Figure 1A, B, E), with no effect on IPSC amplitude (49.6±10.1 and 46.0±10.9 pA, baseline and AM251, respectively, Figure 1C). AM251 produced no change in IPSC kinetics (Figure 1D). In addition, AM251 did not significantly affect the frequency of excitatory postsynaptic currents (EPSCs; 0.5±0.1 and 0.63±0.12 Hz, baseline and AM251, respectively), or EPSC amplitude (22.4±3.6 and 23.6±3.1pA, baseline and AM251 respectively, n = 7, data not shown). Conversely, the CB1-R agonist WIN 55,212-2 decreased the frequency of IPSCs onto POMC neurons (38%, 1.3±0.5 and 0.8±0.3 Hz, baseline and WIN 55,212-2 respectively, n = 5, p<0.05) with no effect on amplitude (38.6±9.2 and 37.6±9.2 pA, baseline and WIN 55,212-2, respectively, data not shown). Curiously, WIN 55,212-2 also decreased the frequency of EPSCs onto POMC neurons (64%, 2.5±0.8 and 0.9±0.4 Hz, baseline and WIN 55,212-2, respectively; n = 4, p<0.05), with no effect on amplitude (25.2±4.1 and 22.2±3.5 pA, baseline and WIN 55, 212-2, respectively, data not shown). Our findings that a CB1-R antagonist inhibits the activity of anorectic POMC neurons are consistent with those of Hentges et al. (2005) [8]. However, these findings are inconsistent with the known anorectic effects of CB1-R antagonists. In addition, the CB1-R agonist decreased the frequency of both inhibitory and excitatory currents onto POMC cells, suggesting a negligible effect on overall POMC neuronal activity. Thus, it seems unlikely that cannabinoids' dramatic effects on feeding can be explained on the basis of their effects on POMC neurons alone. We further measured the release of α-MSH from hypothalamic explants incubated with AM251 or WIN 55, 212-2 to test the sensitivity of POMC neurons to cannabinoids. There was no significant difference in α-MSH levels in explants incubated with AM251 or WIN 55, 212-2 compared to explants incubated with aCSF (Figure S1). In light of above results, immunohistochemical staining for c-Fos activation and POMC-GFP expression were analyzed in the ARH of IP AM251 and saline treated animals. c-Fos expression was increased significantly by AM251 between saline and treated mice (p = 0.0012) (Figure S2). POMC-EGFP cell counts are not significantly different between saline and treatment groups. There was a trend towards an increase in c-Fos and POMC-EGFP co-localization in vehicle (n = 6; 6%) and AM251 treated group (n = 6; 13%; p>0.05, paired t test) but this was not significant and lends support to the electrophysiological and radioimmunoassay data above, that is, the activation observed in the ARH with AM251 was not robust.

### Cannabinoid modulators similarly regulate food intake in A^y^ and WT mice

**Figure 2:**
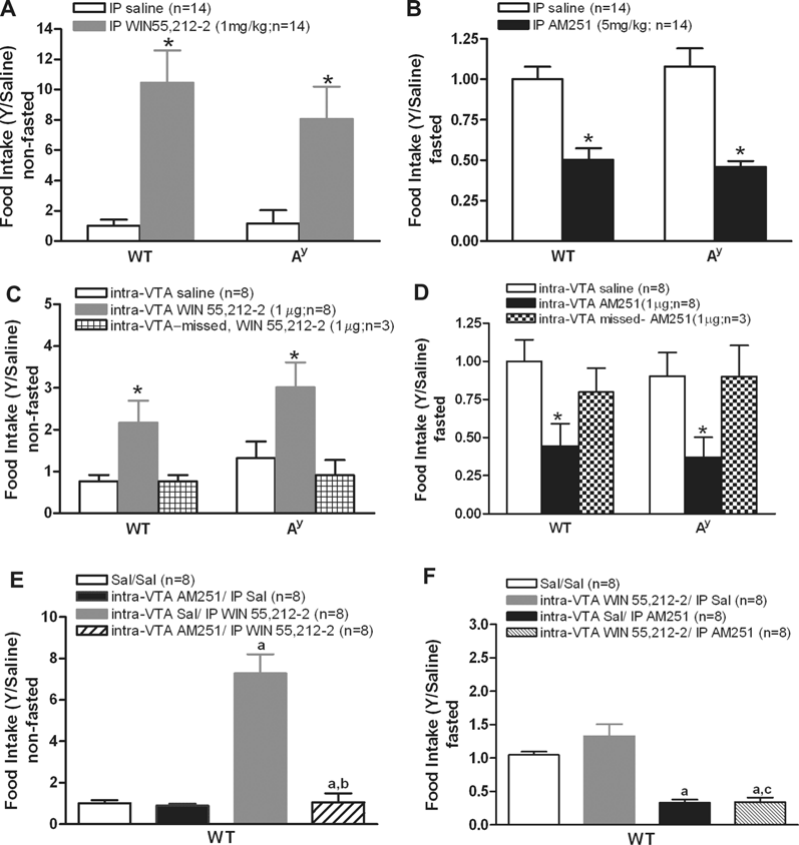
Effects of cannabinoid agonist and antagonist on food intake. *A*, IP WIN 55,212-2 increased 1-hour food intake in both A^y^ and WT mice; *B*, IP AM251 decreased food intake in both A^y^ and WT mice; *C*, Intra-VTA WIN 55,212-2 increased food intake and *D* intra-VTA AM251 decreased food intake in both genotypes. Food intake data presented as fraction of control (*p <0.05, when compared with saline). Effect of intra-VTA AM251 on feeding induced by peripheral WIN 55,212-2; *E*, Intra-VTA AM251 blocked feeding induced by IP WIN 55, 212-2 in WT mice (a: p <0.001 compared to saline/saline (Sal/Sal); b: p<0.001 compared to intra-VTA saline/IP WIN 55,212-2 treatment group); *F*, IP AM251 causes similar reduction in response to intra-VTA WIN 55,212-2 administration (a; p<0.001 compared to (Sal/Sal); c:.p<0.001 compared to intra-VTA WIN 55,212-2/IP AM251 treatment group).

The role of the melanocortin circuitry in cannabinoid-modulated feeding was next tested by assessing differences in feeding behavior between wildtype (WT) and A^y^ mice. Intraperitoneal administration of the cannabinoid agonist WIN 55,212-2 (1 mg/kg) similarly increased 1-hour food intake by 80% in both A^y^ and WT mice (F_(3,51)_  = 9.40, P<0.0001; Figure 2A).This effect was transient, no longer significant by the 4, 8, or 24 hour timepoints. At doses greater than 1 mg/kg (2.5 and 5 mg/kg), mice were partially supine and inactive for at least 2 hours postinjection, compared to saline-injected mice (data not shown). In order to investigate feeding inhibition by the cannabinoid antagonist, mice were fasted overnight. Intraperitoneal (IP) administration of AM251 significantly decreased 1-hour food intake in both A^y^ and WT mice compared to saline (F_(3,51)_  = 16.53, P<0.0001; Figure 2B). Similar to WIN 55,212-2, the effects of AM251 were transient; the reduction in food intake was absent by 8 h postinjection. Likewise, both genotypes decreased fasting-induced re-feeding 47% in response to AM251 (5 mg/kg, Figure 2B).

Central administration of the cannabinoid agonist and antagonist produced comparable responses to those of peripheral administration. Intra-VTA WIN 55,212-2 administration increased 1-hour food intake in nonfasted A^y^ and WT mice (F_(3,31)_  = 3.98, P = 0.016; Figure 2C) and AM251 decreased 1-hour food intake in fasted A^y^ and WT mice (F_(3,27)_  = 3.98, P = 0.017; Figure 2D) compared to saline treatment and intra-VTA missed treated groups. The fact that A^y^ mice respond normally to peripheral and central AM251 indicates that endogenous antagonism of MC3/4 receptors (in A^y^ mice) does not disrupt feeding responses to cannabinoids.

To further investigate the role of the VTA as a potential mediator of cannabinoid-induced feeding, we assessed whether disrupting cannabinoid signaling in the VTA would prevent systemic cannabinoid agonist from inducing hyperphagia. Intra-VTA AM251 prevented the increase in food intake induced by IP WIN 55,212-2 in non-fasted WT mice (F_(3,27)_  = 18.67, P<0.0001, Figure 2E), demonstrating that peripheral cannabinoid-induced feeding requires CB1-R activation in the VTA. In contrast in fasted WT mice, intra-VTA WIN 55,212-2 did not block the feeding inhibition induced by IP AM251 (F_(3,25)_ = 23.45, P<0.0001), Figure 2F).

### Peripheral administration of WIN 55,212-2 or AM251 reveals similar central c-Fos staining in A^y^ and WT mice

We performed c-Fos immunohistochemistry to determine the activation of central circuitry after peripheral (IP) administration of WIN 55,212-2 and AM251 in A^y^ and WT mice (Table 1, Figure S3). c-Fos expression was analyzed in the NAc, ARH, PVH, LH, CeA, VTA, LC, PBN and NTS. The CB1-R agonist WIN 55,212-2 substantially increased c-Fos IR in the LC, PBN and NTS, and modestly increased c-Fos IR in forebrain structures such as the PVH and CeA in both A^y^ and WT mice. Intraperitoneal AM251 produced significant c-Fos IR in the ARH, PVH, LH, CeA and the shell of the NAc, and also in hindbrain regions such as the LC, PBN and NTS in A^y^ and WT mice. Peripheral AM251 also increased c-Fos IR in regions of the brain associated with reward, such as the shell of the NAc and CeA. The core of the NaC expressed low c-Fos IR with peripheral WIN 55,212 or AM251 (data not shown). Peripheral WIN 55,212-2 or AM251 did not induce significantly different patterns of cellular activation between the genotypes, indicating that the cannabinoid agonist and antagonist activate similar brain regions despite the absence of a functional melanocortin system. These data suggest that cannabinoids may produce their effects on feeding by activating both homeostatic regulators of feeding such as the LH and PVH and reward-based areas such as the CeA and NAc that integrate and relay various properties of food to drive feeding behavior.

**Table 1:** Effect of peripheral (IP) and central (intra-VTA) WIN 55,212-2 and IP AM251 on c-Fos IR in A^y^ and WT mice.

		NAc shell	ARH	PVH	LH	DMH	VMH	CeA	VTA	LC	PBN	NTS
**IP Administration**												
**WT**	Saline	0.9±0.5	1±0.5	0.6±0.3	2.2±1.3			3.5±1.5	1.6±0.7	3.4±2.3	2.5±1.3	4±1.2
	WIN 55,212-2	7±1.6	8±1.8	15±3.9***	9.8±2.4			22±2.5***	5.7±1.4	31±3.8***	44±9.5***	30±3.5***
***A^y^***	Saline	3±1.3	1±0.4	3.6±1.8	1±0.4			1.4±0.9	0.7±0.5	7±1.4	1.3±0.6	4.2±0.8
	WIN 55,212-2	11±3.1	7±1.7	9±4	4±1.9			30±3.9***	4.2±1.2	22±1.4**	34±5.5***	37 ±4.7 ***
**WT**	Saline	0.7±0.4	5±0.7	10±1.4	16±1.8			7±2.6	2±0.6	7±3.2	0.8±0.4	7±2.5
	AM251	17±4.3***	14±2.3*	14±2.7	23±3.5			22±4.6**	9±1.7	22±1.5*	30±9.1***	16±6.3
***A^y^***	Saline	18±6	2±0.4	7±1.2	11±1.6			3±1.6	1±0.3	4±0.4	1.5±0.5	10±4
	AM251	36±3.8***†††	12±2**	21±2.7***	29±3***			9±2.4††	9±2.2	18±0.5	63±6.2***†††	33±3.8***††
**Intra-VTA Administration**												
**WT**	Saline	2±1.5	4±2.7	7±3.1	7±4.1	3±1	4±0.5	4±2	2±0.7	2±0.3	2±0.8	7±2.2
	WIN 55,212-2	22±2.8**	11±0.8	19±4.6	27±4.9	6±0.1	8±0.5	8±1.4	5±0.4	17±3.4**	21±3.6**	16±3.8
***A^y^***	Saline	3±1.3	6±2.2	3±0.7	4±2	6±3.5	4.5±3.5	4±1.5	2±0.8	9.4±3.9	6±2.1	5±1.9
	WIN 55,212-2	12±3.5	9±1.3	28±5.6***	24±1.7	12±0.5	14±1.5	15±3.5	6±1.0	37±5.3***†††	38±2.5*** ††	33±5.2***†††
**WT**	Saline	4±1.4	5±2.1	13±4.7	9±4	12±4.5	13±4.1	3±0.5	8±0.8	1±1	1±1	3±1.5
	AM251	6±1.2	10±2.6	25±5.5	25±2.7*	50±5.5**	39±3.1**	12±2.5	8±0.9	18±3.2	8±4.6	7±2
***A^y^***	Saline	5±2.1	9±2.9	10±7.1	5±0.7	7±2.4	10±3.7	3±0.75	4±1.7	14±5.6	14±4	9 ±3 .7
	AM251	8±3.2	14±3.5	16±4.1	16±5	39±1.0**	24±7.2	11±1.5	6±0.9	26±3.2	22±3.9	12±2.3

Male A^y^ and WT mice were perfused with 4% paraformaldehyde 90 min after IP injection with saline-DMSO (n = 3), WIN 55,212-2 (1 mg/kg;n = 3) or AM251 (5 mg/kg; n = 4). Another group of male A^y^ and WT mice was perfused 90 min after intra-VTA injection with saline-DMSO (n = 3), WIN 55,212-2 (1 µg; n = 3) or AM251 (1 µg; n = 4). Immunohistochemical staining for c-Fos expression in nucleus accumbens, NAc (shell), arcuate nucleus (ARH), paraventricular nucleus (PVH), lateral hypothalamus (LH), dorsomedial hypothalamus (DMH), ventromedial hypothalamus (VMH), central amygdala (CeA), ventral tegmental area (VTA), locus coerulus (LC), parabrachial nucleus (PBN) and nucleus of the solitary tract (NTS). Results are expressed as mean±SEM.^*^ p <0.05, ^**^ p <0.01, ^***^ p <0.001 compared to saline; ^†^ represent statistical comparisons between WT and A^y^ mice; ^†^ p <0.05,^††^ p <0.01, ^†††^ p <0.001.

### Intra-VTA WIN 55,212-2 produces significant c-Fos IR in hindbrain and forebrain areas involved in feeding and reward in A^y^ and WT mice

To further examine the potential role of reward circuitry in cannabinoid effects, we injected cannabinoids directly into the VTA (Table 1, Figure S4). Immunohistochemical staining for c-Fos expression was analyzed in the NAc (shell and core), bed nucleus of the stria terminalis (BST), ARH, PVH, LH, DMH, ventromedial hypothalamus (VMH), CeA, VTA, LC, PBN and NTS. There were no significant differences in c-Fos IR between central WIN 55212-2 or AM251 treatment between genotypes. Similar to peripheral administration, central (intra-VTA) WIN 55,212-2 increased c-Fos IR in the LC, PBN and NTS in both genotypes, suggesting that the agonist either directly activates neurons or removes inhibitory tone onto cells within these areas. Compared with peripheral administration, intra-VTA AM251 produced more staining in hypothalamic structures such as the LH, DMH, and VMH, possibly indicating downstream activation of these regions from the VTA. Central AM251 produced no significant activation in the LC, PBN, and NTS. Interestingly, there was a trend for increased c-Fos staining in the LH with intra-VTA administration of both the cannabinoid agonist and antagonist (Table 1), suggesting that distinct cell phenotypes, possibly orexin and melanin-concentrating hormone (MCH), may be activated.

**Figure 3:**
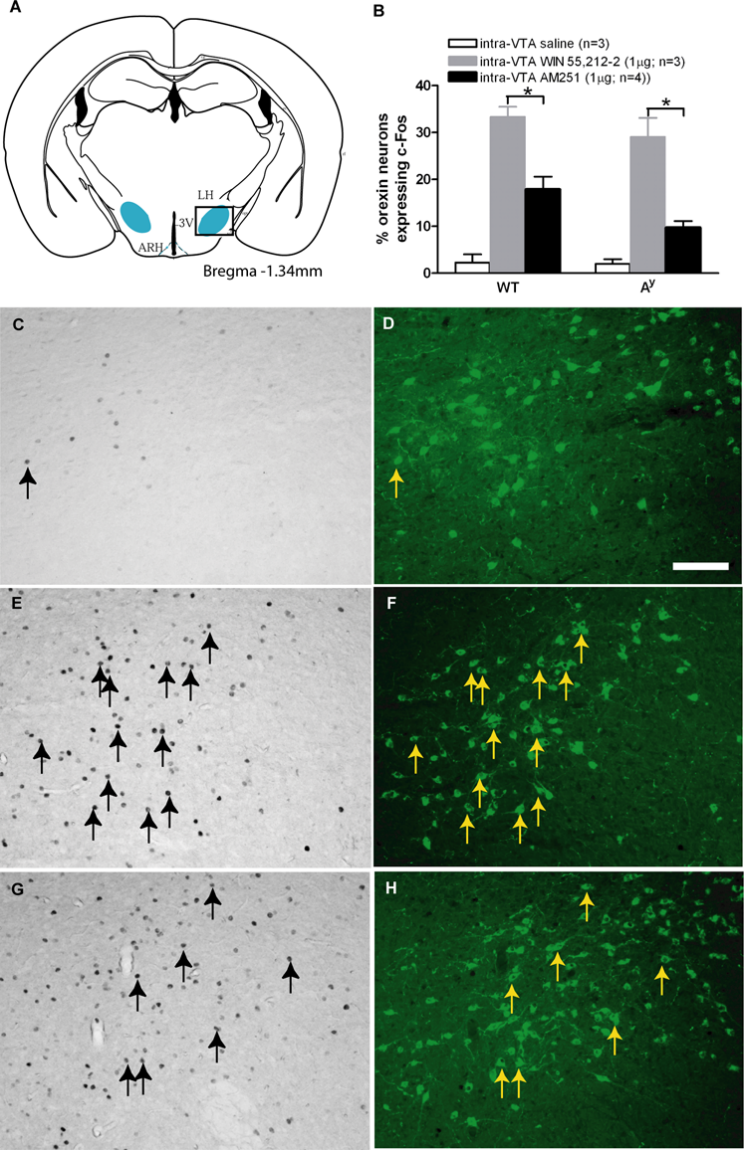
c-Fos and orexin co-localization in the LH in A^y^ and WT mice after intra-VTA injection with saline, WIN55,212-2 or AM251. *A*, Coronal cross-section showing where manual counts of positively stained neurons were made (blue shaded region). c-Fos staining indicated in brightfield images and orexin neurons shown in fluorescence (FITC-green). Arrows indicate co-localised neurons for c-Fos (black arrows) and orexin (yellow arrows) in WT mice. *B, G, H*, Small percentage of orexin neurons co-localized with c-Fos activation in the LH with central AM251 administration. *B, E, F,* A greater percentage of c-Fos positive orexin neurons was found with central WIN 55,212-2 treatment compared to *B, C, D,* saline treatment. Scale bar, 100 µm.

We further carried out double-labeling for c-Fos and orexin or MCH. Central CB1-R agonist administration produced a significant percentage of c-Fos positive orexin neurons in the LH (33.3±2.2% WT; 29.1±4.1% A^y^ , p<0.05, Figure 3B, E, F), compared to saline treatment (1.2±0.7% WT; 4.6±2.3% A^y^, Figure 3B, C, D). After central CB1-R antagonist administration, a smaller percentage of orexin neurons co-localized with c-Fos IR in the LH (18.5±2.7% WT; 9.3±1.4% A^y^, p<0.05, Figure 3B, G, H), compared to saline treatment. Co-localization of c-Fos with MCH was not observed with either central CB1-R antagonist or agonist treatment in WT or A^y^ mice (data not shown). Intra-VTA cannabinoids activate appetite-stimulating orexin neurons and not MCH neurons, suggesting a specific delineation of roles with respect to regulation of cannabinoid effects.

### AM251 increases evoked dopamine release in coronal nucleus accumbens slices

**Figure 4:**
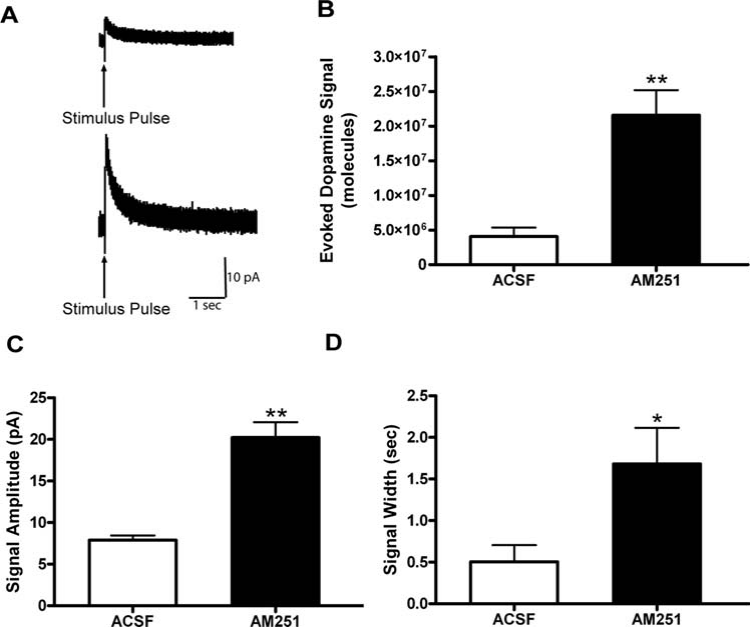
Effect of AM251 on electrically stimulated dopamine release from the NAc shell. *A*, Representative amperometric traces of electrical stimulation-evoked dopamine release in the nucleus accumbens shell from coronal slices of Sprague Dawley rats. A 0.5 mA electrical pulse lasting 2msec (arrow) was used as a stimulant with an interstimulus interval of 5 min to allow for full recovery. Data was recorded at 50 kHz and filtered at 1 kHz. The top trace is representative of the ACSF group and the bottom trace is representative of the evoked dopamine response after a 30 min 3 µM AM251 wash. *B*, The mean number of evoked dopamine molecules significantly increased from 3.8×10^6^±1.2×10^6^ in ACSF to 21.6×10^6^±3.6×10^6^ after 3 µM AM251 (n =  11 stimulations in ACSF, n =  15 stimulations after 3 µM AM251 in 3 slices per group from 3 animals, **p<0.01 by 1-way ANOVA). *C*, The mean stimulated signal amplitude increased significantly from 7.9±0.6 pA in ACSF to 20.2±1.8 pA in 3 µM AM251 (**p<0.01). *D*, The mean stimulated signal width increased significantly from 0.5±0.2 seconds in ACSF to 1.6±0.4 seconds in 3 µM AM251 (*p<0.05). All values are expressed as mean±SEM.

Because the reward circuitry is more likely than the melanocortin circuitry to be responsible for the effects of cannabinoids on feeding [18]–[21], we tested how cannabinoids affect dopamine release from the VTA. We directly assessed evoked dopamine release from NAc terminals in acute slice preparations from rats using electrochemical methods [22] (Figure 4). A carbon fiber electrode was inserted into the NAc shell to collect amperometric recordings from dopaminergic axons arising from the VTA. The current produced by the electrochemical oxidation of extracellular dopamine measures dopamine release kinetics in real time. As shown in Figure 4A, electrical stimulation by an adjacent bipolar stimulating electrode increased dopamine release from NAc terminals. After bath application with the CB1-R antagonist AM251 (3 µM), electrically evoked dopamine release increased significantly (3.8×10^6^±1.2×10^6^ and 21.6×10^6^±3.6×10^6^ molecules, ACSF and AM251, respectively, F_(1,24)_ = 13.35, P =  0.0005; Figure 4B). AM251 also significantly increased evoked signal amplitude (20.2±1.8 and 7.9±0.6 pA, F_(1,24)_ = 31.07, P<0.0001) and evoked signal width (1.6±0.4 and 0.5±0.2 sec, AM251 and ACSF respectively, F_(1,24)_ = 5.09, P = 0.033; Figure 4C, D). This shows that AM251 is regulating stimulated DA release from VTA terminals in the NAc.

## Discussion

Cannabinoid effects on feeding are unaffected by the absence of a functional melanocortin system–no differences were observed between WT and A^y^ mice in the feeding responses to either a CB1-R agonist or antagonist. Regional brain activation by either central (intra-VTA) or peripheral (IP) cannabinoid administration was nearly indistinguishable between genotypes. Our main findings demonstrate that functional central melanocortin circuitry is not required for cannabinoid-induced modulations in feeding behavior, but rather that activation of dopaminergic reward circuitry is involved in cannabinoid's effect on feeding.

Our observation that a CB1-R antagonist related to Rimonabant increased inhibitory synaptic currents onto identified POMC cells *in vitro* is contrary to what would be predicted based on the known anorectic role of POMC neurons and the demonstrated effects of cannabinoid agonists and antagonists on feeding. If feeding inhibition induced by the CB1-R antagonist was mediated by increasing α-MSH release (therefore stimulating MC4-R), we would predict that AM251 would produce either a decrease in IPSC frequency or an increase in EPSC frequency onto POMC cells–which was not observed. Additionally, the CB1-R agonist WIN 55,212-2 reduced both spontaneous inhibitory and excitatory inputs onto POMC neurons, suggesting that cannabinoids may have little net effect on the excitability of POMC neurons. Our findings are consistent with those of Ho and colleagues in which a dose-dependent decrease in EPSC frequency was found with WIN 55,212-2 onto POMC neurons in the guinea pig ARH [23]. In contrast with their findings, we conclude that the net effect of WIN 55,212-2 onto arcuate POMC neurons is likely negligible since the agonist also reduces the frequency of spontaneous IPSCs onto these neurons. We found that cannabinoids did not effect α-MSH release from POMC neurons, and co-localization of c-Fos and POMC-EGFP neurons in response to IP AM251 was marginal. Pathways other than the melanocortin system are likely responsible for feeding effects induced by cannabinoids. This conclusion is corroborated by our feeding studies in which cannabinoids are equally efficacious in WT and A^y^ mice.

Rimonabant and AM251 reliably decrease food intake and body weight in obese *fa/fa* rats, and both *ob/ob* and A^y^ mice [24], [25], often with greater effects than in lean littermates. In rats, Verty et al. (2004) demonstrated that central THC (a CB1-R agonist) increased food intake and central α-MSH decreased food intake, but that THC-induced feeding was not inhibited by α-MSH [26]. However, Verty et al. (2004) further found that a CB1-R antagonist (SR 141716) reduced food intake induced by a central MC4-R antagonist (JKC-363). In contrast, we demonstrate that AM251 attenuates food consumption in A^y^ mice (a functional model of MC4-R antagonism) the same amount as WT mice. Signalling through MC4R is abolished in both A^y^ and MC4R knockout mice; as with all genetic modifications, developmental compensation cannot be excluded. There are at least three likely reasons for this discrepancy between our data and that of Verty et al. (2004). The first is the mechanism of blockade of MC4 receptors. In the A^y^ mice, Agouti protein binds to the MC4-R and stabilizes it in its inactive conformation, rather than simply blocking the receptor in a competitive fashion [27]. Second, to ensure the VTA would be activated by central cannabinoids, we injected agents directly into the VTA, whereas Verty et al.(2004) administered agents into the lateral ventricle. Third, different species used for investigation of the circuitry may also account for the different results.

In humans, cannabinoids are involved in food orosensory-based reward. Subjects increase consumption of highly palatable foods in response to marijuana [28]. In animal models, it is well established that activation of reward pathways provides both a strong influence on food choice [29], [30] and feeding behavior [31], associated with an increase in dopamine release in the NAc [32]. Here, we report that peripheral WIN 55,212-2 and AM251 induced c-Fos staining in structures such as the NAc shell, PVH, LH, CeA, LC, PBN, and NTS in mice. Little or no c-Fos IR was observed in the prefrontal cortex, caudate putamen, BST, or VTA-areas of AM251-induced c-Fos IR in rats [33]–[35]. Our results indicate no clear genotypic differences in response to peripheral WIN 55, 212-2 or AM251 treatment. We observed strong c-Fos expression induced by IP AM251 in the NAc shell, LH, CeA, and NTS, suggesting that these nuclei form a functional circuit through which cannabinoids mediate changes in food intake. Cannabinoids may produce their effects on feeding by recruiting various anatomical sites-inside and outside the hypothalamus-that integrate and relay gustatory, sensory and reward properties of food [36].

In our study, intra-VTA WIN 55, 212-2 injection increased c-Fos activation in the PVH, LH, PBN, and NTS. Intra-VTA AM251 significantly increased Fos-IR in several hypothalamic nuclei, including DMH and VMH, which was not observed with peripheral AM251, suggesting that cellular activation within these regions was due to downstream activation of the VTA. The DMH and VMH are targets of converging orexigenic and anorexigenic pathways [37], and are known to regulate food intake by influencing metabolic, hormonal, and endocrine responses relating to the nutritional state of the organism [38]. Neuronal DMH activation leads to increased sympathoexcitation and specifically brown adipose tissue (BAT) thermogenesis [39]. Furthermore, activation of neurons in the LH increase sympathetic nerve drive to BAT, in a pathway dependent on activity from neurons in the DMH [40]. VMH administration of anandamide, an endogenous cannabinoid, induces feeding via CB1-R activation [41]. Downstream neuronal activation in both VMH and DMH by intra-VTA AM251 may promote weight loss via simultaneously suppressing appetite (i.e., VMH activation) and by a sympathetically-mediated increase in metabolic activity (i.e., DMH activation).

Lesion studies have also indicated that the LH regulates ingestive behavior [42]. Within the LH, two neuropeptides, MCH and orexin, are localized to distinct neuronal populations. Orexin neurons in the LH project to the VTA and orexin increases the firing rate of VTA neurons [43]. Endogenous orexin signaling to VTA dopaminergic neurons is critical for the expression of motivated behaviors [44]. Cannabinoids have been shown to modulate the electrophysiological activity of orexin and MCH neurons [45], [46]. Consistent with orexin's recognized appetite-stimulating effect [47], we found a significant amount of co-localization of orexin and c-Fos (30%) after central WIN 55, 212-2 administration. However, these results are inconsistent with those of Huang et al. (2007). In acute slice preparations of mouse hypothalamus, Huang et al. (2007) found that WIN 55,212-2 inhibited orexin neurons via decreasing excitatory synaptic inputs. We observed a small percentage of orexin neurons (10%) that co-localized with c-Fos in the LH after central AM251 administration, consistent with Huang et al's (2007) finding that AM251 increased the activity of orexin neurons. In contrast with Huang et al. (2007) and Jo et al. (2005) in vitro studies, MCH IR neurons were not activated by in vivo central WIN 55,212-2 administration. In vitro slice techniques are more sensitive than c-Fos IR, and it is possible that we were unable to detect activation of MCH neurons. Our detection of orexin neuron activation by WIN 55,212-2 may reflect that the drug was given in awake cannulated mice versus direct application in acute slice preparations and we used doses that did not alter non-feeding behavior. At any rate, we speculate that the two groups of orexigenic neurons may possibly play distinct roles with regards to modulation of cannabinoid-induced effects. Clearly, orexin from the LH appears to provide a feedback system into the VTA, possibly activating dopaminergic systems.

Our findings show that the CB1-R antagonist AM251 increased electrically evoked release of DA in NAc, in acute slices. It has been shown that CB1-R antagonists in the VTA decrease activity of DA neurons induced by a CB1-R agonist [11], [12]. Thus, the effects of CB1-R antagonist in our preparation are complex to interpret, especially in the absence of a clear paradigm of how DA signaling regulates reward and satiety.

At the behavioral level, AM251 suppresses food intake in mice [48]. Increased dopamine in the NAc during feeding and at the end of the meal has been shown as a signal for meal termination and satiation [49]. Thus, increased dopamine release in the NAc may contribute to the anorexic effect of AM251.

Rats with high motivation to initiate feeding (underweight food-deprived rats and dietary obese rats) have low, not high, basal extracellular dopamine in the NAc [50]–[52]. While dopamine is essential for feeding, excess dopamine (as, for example, induced pharmacologically by AM251 in our study) inhibits feeding. This has been previously demonstrated by (a) using amphetamine to flood synapses with dopamine [50], (b) preventing dopamine uptake with cocaine or other dopamine transporter inhibitors [51], or (c) administering nonspecific dopamine receptor agonists (apomorphine) [53]. All of these drugs are appetite suppressants and linked to higher dopamine levels in the synapse.

In awake, behaving rats, Cheer *et al.* (2004) [54], using amperometry, demonstrated that the CB1-R agonist WIN 55,212-2 increased the frequency of dopamine transients, while decreasing the amplitude of evoked DA release in the NAc, an effect opposite to and compatible with the increase in amplitude of evoked DA release induced by AM251 in the present study. These data suggest that a sustained increase in DA cell body firing rate eventually depletes the readily available pool of DA-containing vesicles for release in response to electrical stimulation. Therefore, an alternative interpretation of our data is that AM251 acutely decreased the activity of VTA DA neurons without decreasing DA synthesis. This would be accompanied by an increase in DA quantal size which we essentially detected with electrical stimulation of terminals in the NAc. There is not a clear paradigm of how stimulation of DA exocytosis in the NAc regulates food intake. However, it is clear that DA release kinetics can significantly impact DA signaling [55]. Here, we show that intra-VTA CB1-R antagonists inhibit food intake and increase evoked NAc DA release, indicating that DA may well be involved in modulating cannabinoid activity in the reward circuitry but this is not explicitly shown by our data here. Clearly, we do not address issues pertaining to basal DA tone, which is also a critical component of DA signaling in the mesoaccumbal pathways.

Our main result is that cannabinoids exert their effects on feeding via pathways that are independent of the melanocortin system. While other homeostatic hypothalamic regions such as the PVH, LH, VMH, and DMH are likely involved in mediating cannabinoid-induced feeding behavior, one of the key sites for transducing feeding signals–the arcuate nucleus–is likely not critical. Rather, the VTA-NAc axis may well drive feeding behavior induced by cannabinoids in both A^y^ and WT mice. In addition, other reward-based sites such as the CeA and NAc are activated by peripheral cannabinoids in both A^y^ and WT mice. An intra-VTA CB1-R antagonist inhibits food intake, blocks the effect of systemic CB1 agonist, and local slice application of AM251modifies evoked DA release in the NAc, lending support to the hypothesis that activation of mesolimbic pathways is sufficient for many of effects of the cannabinoid antagonists.

## Supporting Information

Figure S1 Effects of WIN 55,212-2 and AM251 on α-MSH release from POMC neurons of the hypothalamus. There was no significant effect on α-MSH release (fmol/ml) from POMC neurons by cannabinoids. All values are expressed as mean±SEM.
(0.14 MB TIF)

Figure S2 Effects of IP AM251 on c-Fos and POMC-EGFP co-localization in POMC-EGFP mice. ARH slices were stained for c-Fos and EGFP expression. c-Fos staining indicated in brightfield images and POMC-EGFP neurons shown in fluorescence (FITC-green). Red arrows indicate c-Fos and POMC-EGFP co-localized cells. A, c-Fos activation in response to IP saline; B, c-Fos and POMC co-localization in response to IP saline; C, c-Fos activation in response to IP AM251 (5 mg/kg); D, c-Fos and POMC co-localization in response to IP AM251. Scale bar, 100 µm. Quantification of immunohistochemical staining for c-Fos and POMC-EGFP co-localization in the ARH. E, c-Fos expression was increased significantly by AM251 between IP saline and AM251 treated groups; F, POMC-EGFP cells counts are not different between saline and AM251; G, Percentage of POMC cells expressing c-Fos IR. All values are expressed as mean±SEM.
(1.73 MB TIF)

Figure S3 Representative photomicrographs showing c-Fos IR in response to IP saline, WIN 55212-2 and AM251 administration in Ay mice. A, B, C, c-Fos activation in response to saline in the NAc, PVH and NTS. D, E, F, Increased c-Fos IR in response to IP WIN 55,212-2. G, H, I, c-Fos IR in response to IP AM251. ac, anterior commissure; 3V, third ventricle, AP, area postrema. Scale bar, 100 µm.
(3.03 MB TIF)

Figure S4 Representative photomicrographs showing c-Fos IR in response to intra-VTA administration of AM251 in the VMH and DMH in WT mice. Inset diagram is representative of VTA cannulation placement with the arrow indicating the tip of the guide cannula; the injector extends 1 mm further for injections. A, c-Fos IR in response to intra-VTA saline. B, c-Fos IR is markedly increased in the VMH and DMH in response to intra-VTA AM251. Scale bar, 100 µm.
(1.04 MB TIF)
