# Supplementary figures and images for: Feeding Induced by Cannabinoids Is Mediated Independently of the Melanocortin System
Source: PLoS One. 2008 May 21;3(5):e2202. doi: 10.1371/journal.pone.0002202 (PMC2386290; doi:10.1371/journal.pone.0002202)

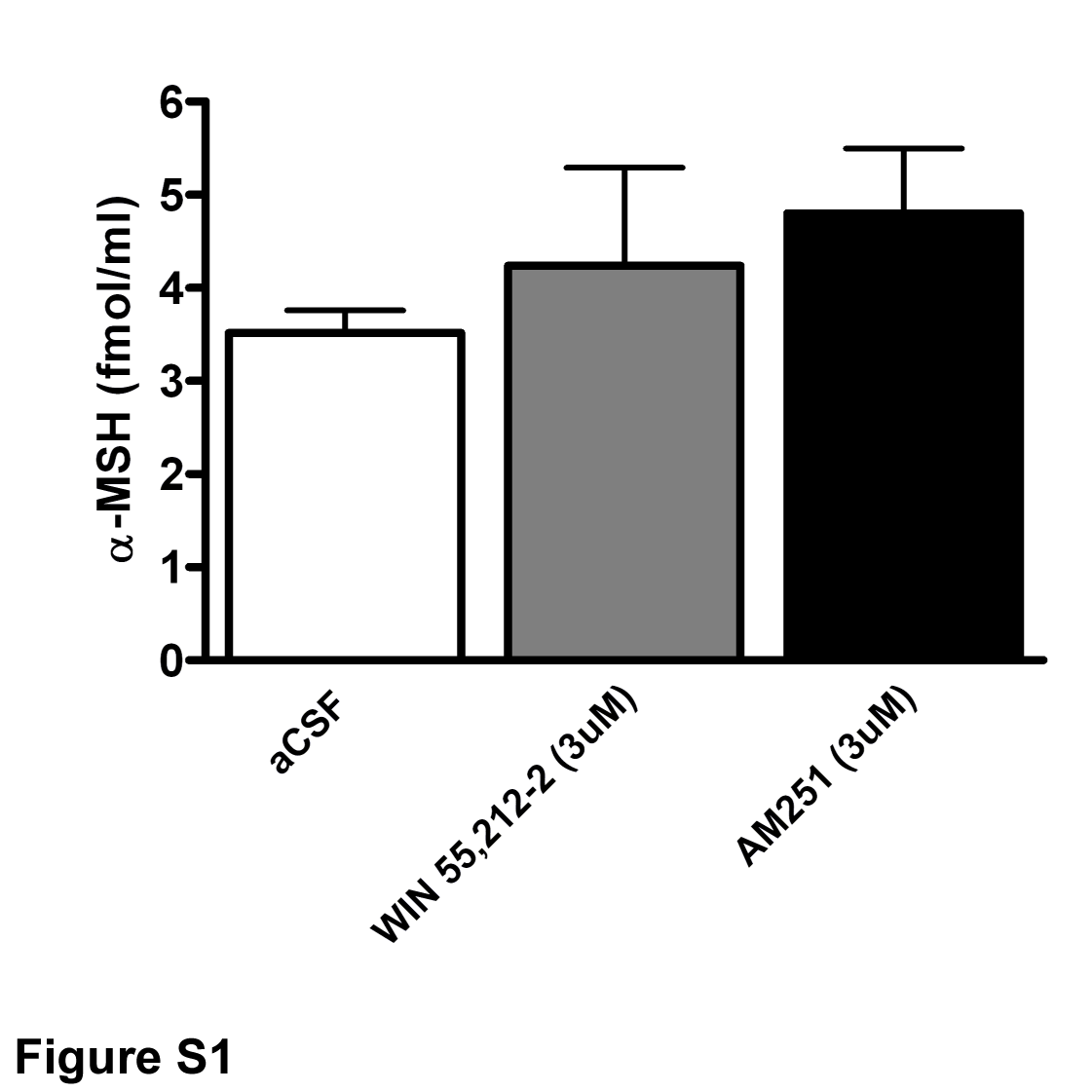

Supplement: Figure S1 — Effects of WIN 55,212-2 and AM251 on α-MSH release from POMC neurons of the hypothalamus. There was no significant effect on α-MSH release (fmol/ml) from POMC neurons by cannabinoids. All values are expressed as mean±SEM. (0.14 MB TIF) [file pone.0002202.s001.tif]

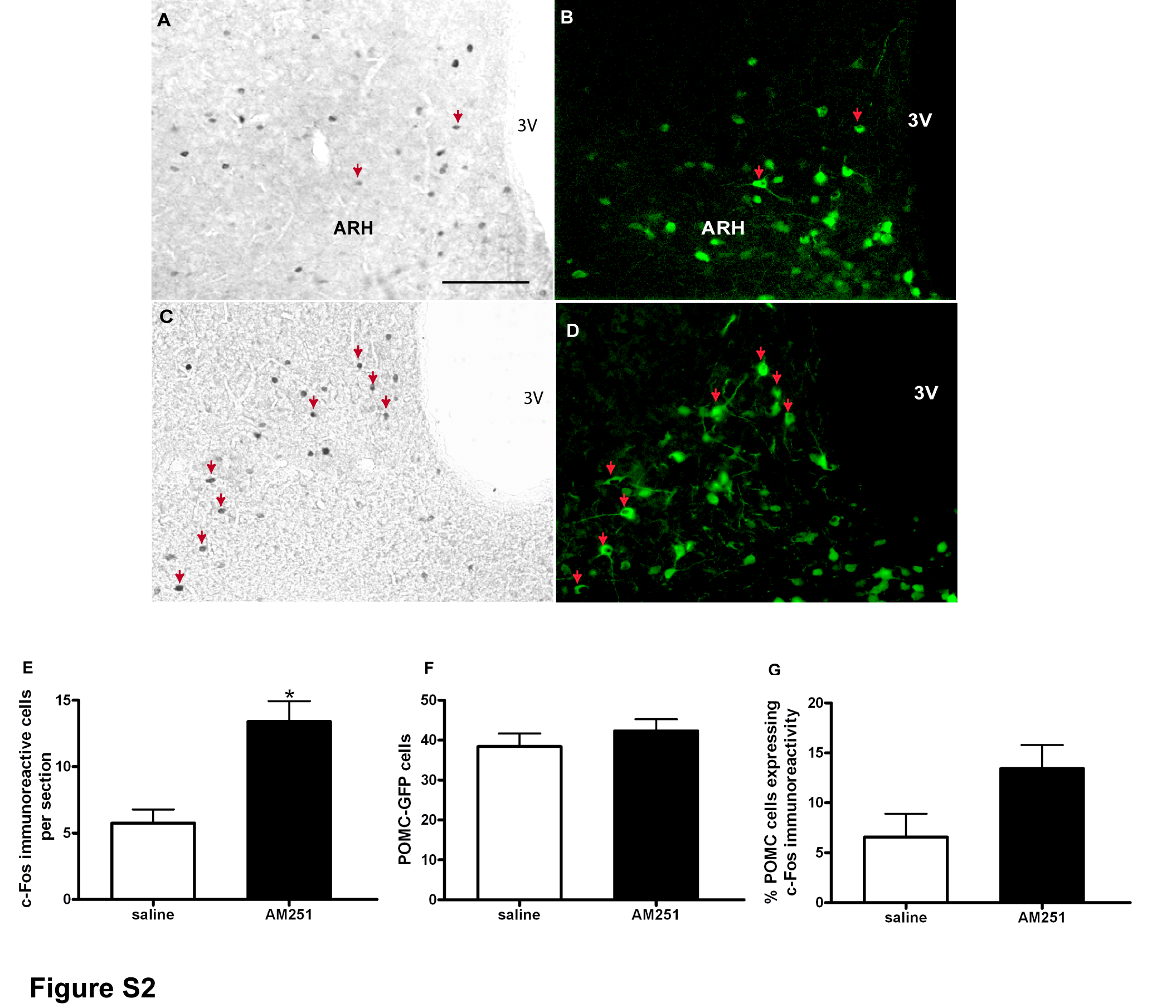

Supplement: Figure S2 — Effects of IP AM251 on c-Fos and POMC-EGFP co-localization in POMC-EGFP mice. ARH slices were stained for c-Fos and EGFP expression. c-Fos staining indicated in brightfield images and POMC-EGFP neurons shown in fluorescence (FITC-green). Red arrows indicate c-Fos and POMC-EGFP co-localized cells. A, c-Fos activation in response to IP saline; B, c-Fos and POMC co-localization in response to IP saline; C, c-Fos activation in response to IP AM251 (5 mg/kg); D, c-Fos and POMC co-localization in response to IP AM251. Scale bar, 100 µm. Quantification of immunohistochemical staining for c-Fos and POMC-EGFP co-localization in the ARH. E, c-Fos expression was increased significantly by AM251 between IP saline and AM251 treated groups; F, POMC-EGFP cells counts are not different between saline and AM251; G, Percentage of POMC cells expressing c-Fos IR. All values are expressed as mean±SEM. (1.73 MB TIF) [file pone.0002202.s002.tif]

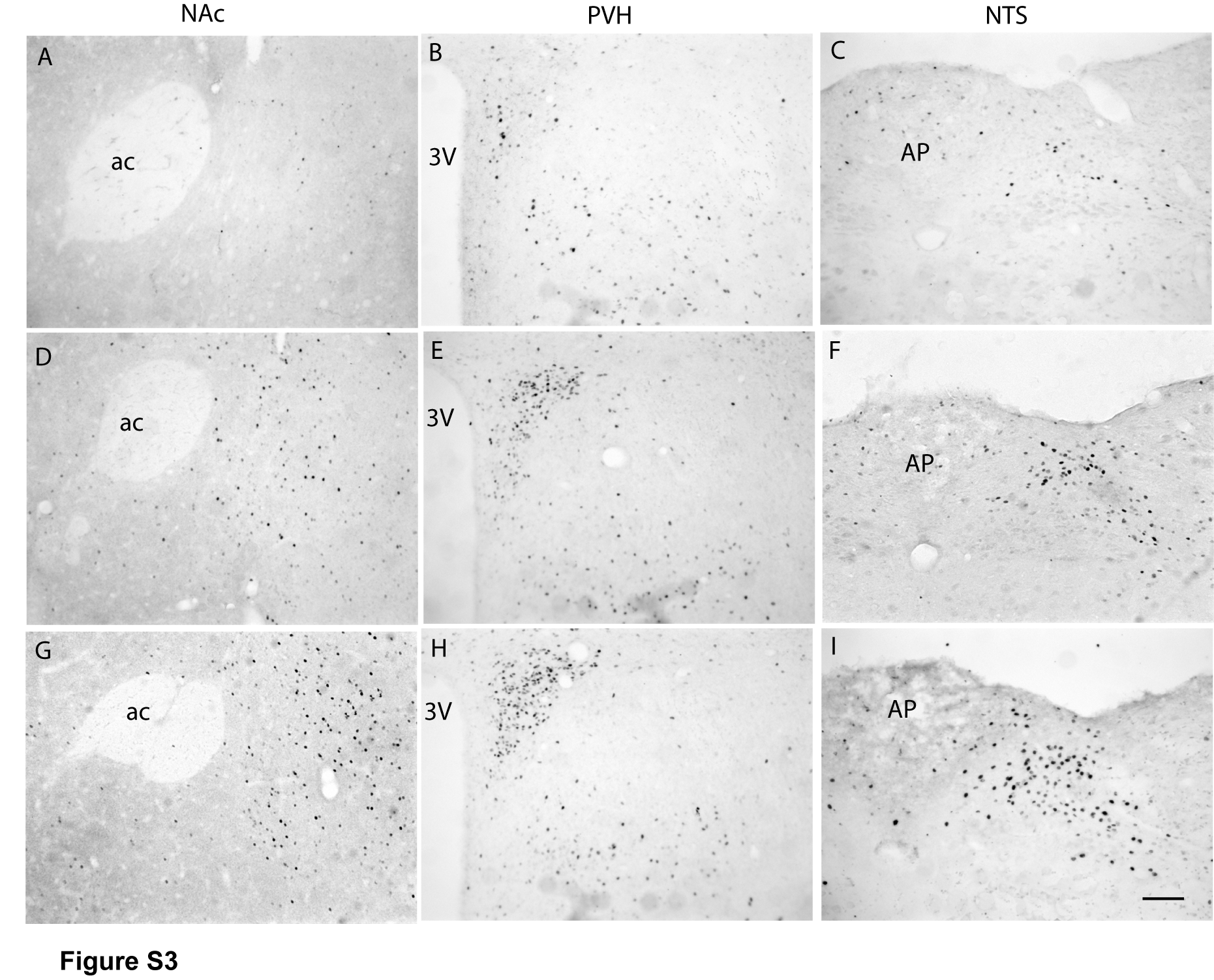

Supplement: Figure S3 — Representative photomicrographs showing c-Fos IR in response to IP saline, WIN 55212-2 and AM251 administration in Ay mice. A, B, C, c-Fos activation in response to saline in the NAc, PVH and NTS. D, E, F, Increased c-Fos IR in response to IP WIN 55,212-2. G, H, I, c-Fos IR in response to IP AM251. ac, anterior commissure; 3V, third ventricle, AP, area postrema. Scale bar, 100 µm. (3.03 MB TIF) [file pone.0002202.s003.tif]

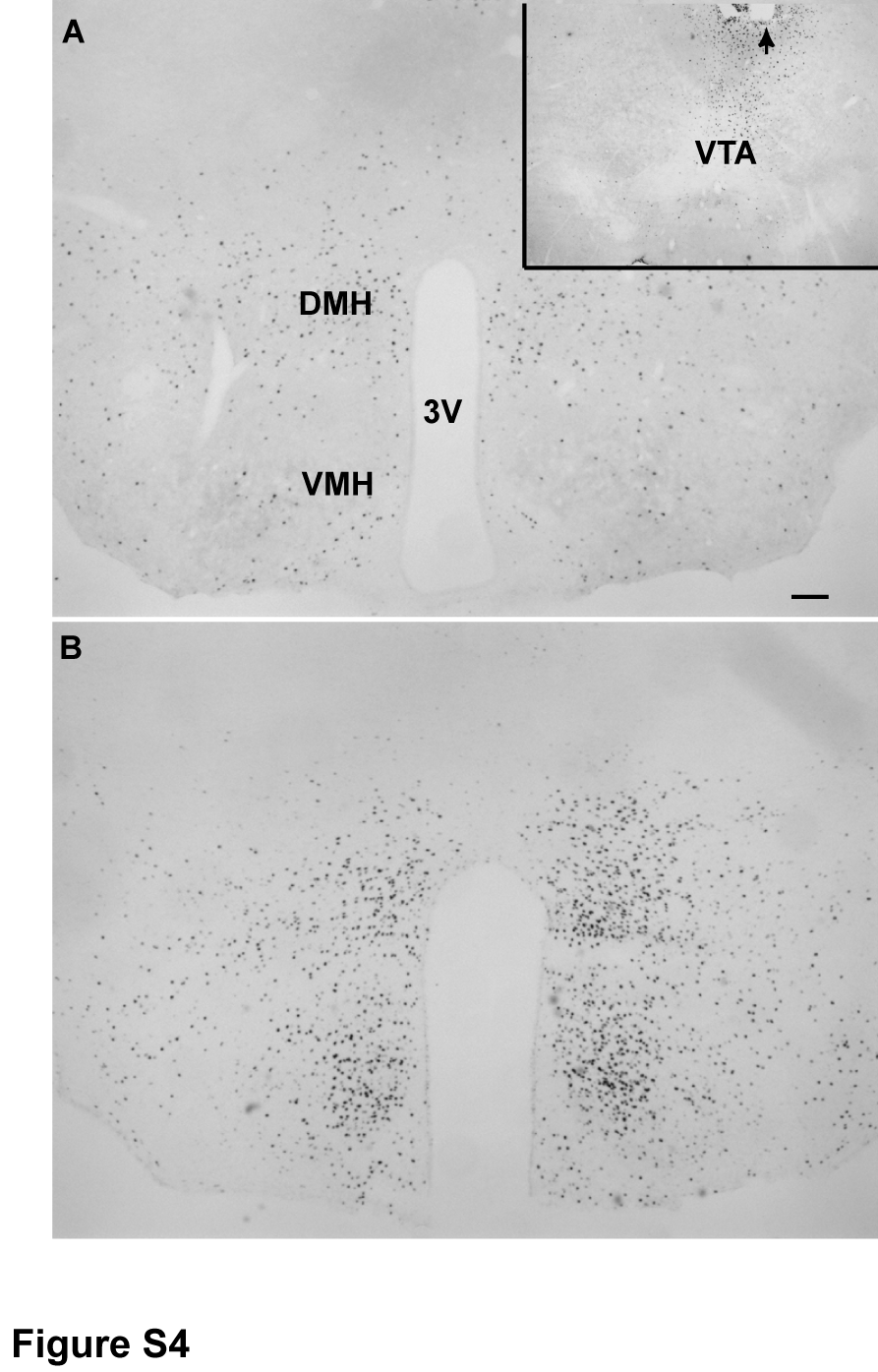

Supplement: Figure S4 — Representative photomicrographs showing c-Fos IR in response to intra-VTA administration of AM251 in the VMH and DMH in WT mice. Inset diagram is representative of VTA cannulation placement with the arrow indicating the tip of the guide cannula; the injector extends 1 mm further for injections. A, c-Fos IR in response to intra-VTA saline. B, c-Fos IR is markedly increased in the VMH and DMH in response to intra-VTA AM251. Scale bar, 100 µm. (1.04 MB TIF) [file pone.0002202.s004.tif]
